# Supplementary material for: Mesenchymal Stem Cell Derived Biocompatible Membrane Vesicles Demonstrate Immunomodulatory Activity Inhibiting Activation and proliferation of Human Mononuclear Cells
Source: Pharmaceutics. 2020 Jun 23;12(6):577. doi: 10.3390/pharmaceutics12060577 (PMC7356506; doi:10.3390/pharmaceutics12060577)
Supplement: Supplementary file 1 [file pharmaceutics-12-00577-s001.pdf]

pharmaceutics

Supplementary Materials: Mesenchymal Stem Cell Derived Biocompatible Membrane Vesicles Demonstrate Immunomodulatory Activity Inhibiting Activation and Proliferation of Human Mononuclear Cells

Marina O. Gomzikova, Sevindzh K. Kletukhina, Sirina V. Kurbangaleeva, Olga A. Neustroeva, Olga S. Vasileva, Ekaterina E. Garanina, Svetlana F. Khaiboullina and Albert A. Rizvanov

Results

1. Impact of CIMVs uptake efficiency on proliferation of lymphocytes

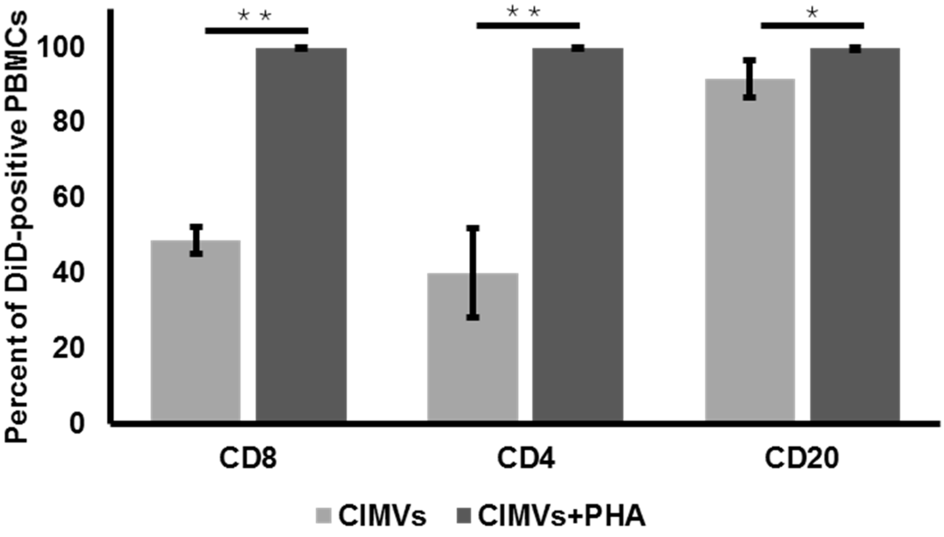

Figure S1. CIMVs-MSCs uptake by lymphocytes. CIMVs-MSCs (10 μg) stained with membrane dye DiD were incubated with PBMCs for 24 h, followed by treatment with PHA (10 μg/mL). PBMCs were analyzed using flow cytometer BD FACS Aria III (BD Bioscience, USA). The data represent mean ± SD. **p*-value ≤ 0.05, ***p*-value ≤ 0.01.

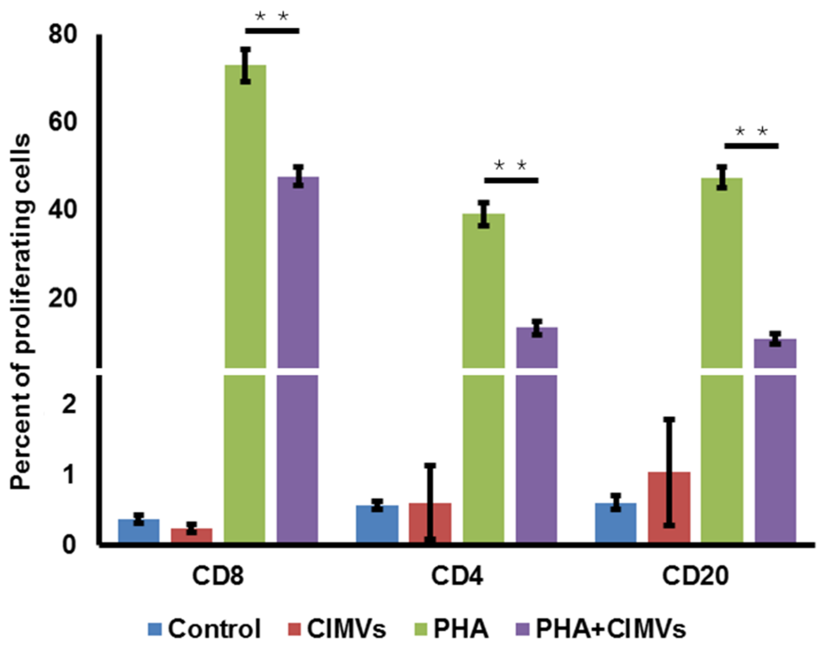

Figure S2. CIMVs-MSCs effect on PHA-induced proliferation of T-cytotoxic (CD8+), T-helper (CD4+), and B-cells (CD20+). Lymphocytes were stained with CFDA SE, followed by incubation with DiD-labeled CIMVs-MSCs for 24 h and treatment with PHA (10 µg/mL). The percent of proliferating cells was evaluated 3 days after PHA incubation. Data represent mean ± SD. **p-value ≤ 0.01.

2. FACS analysis of murine PBMCs: a gating strategy

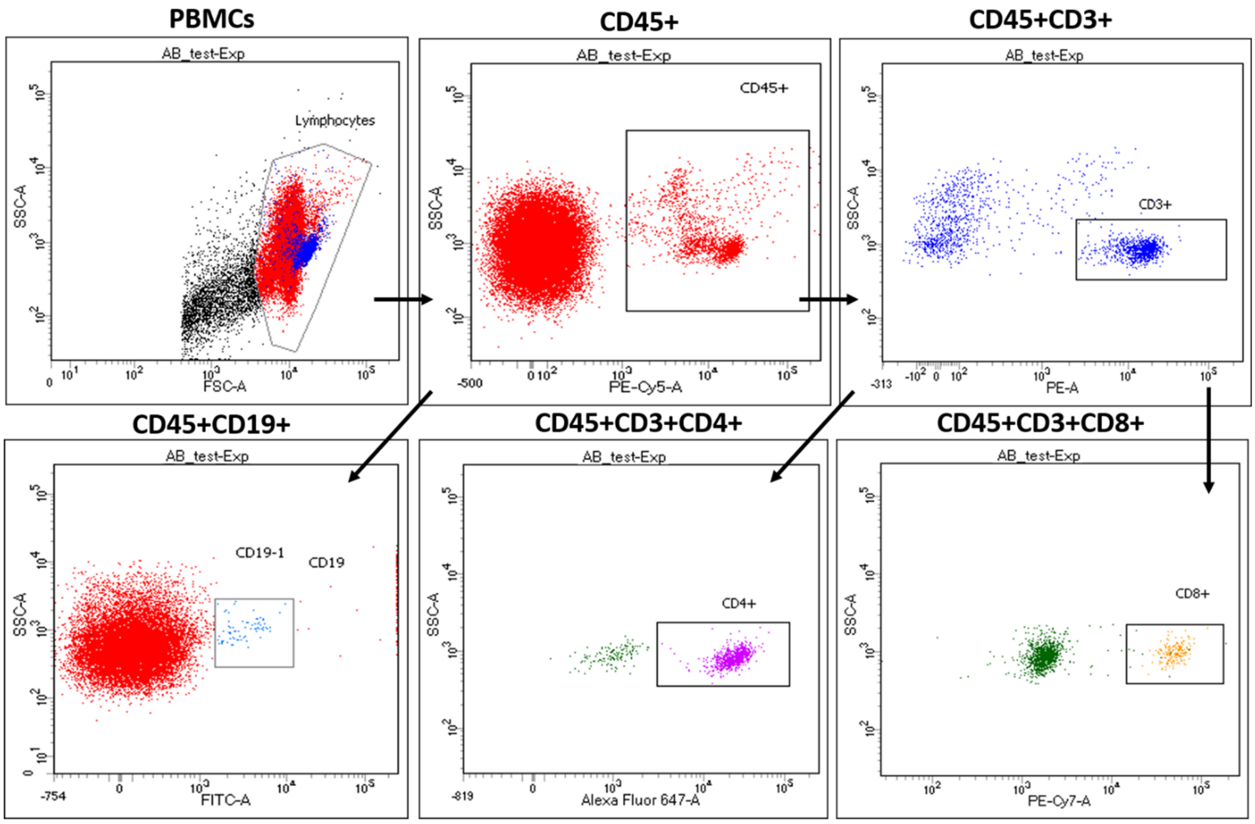

Figure S3. Analysis of murine PBMCs using flow cytometry. PBMCs were incubated with anti-CD45, anti-CD3, anti-CD4, anti-CD19, and anti-CD8 monoclonal antibodies and analyzed using flow cytometer BD FACS Aria III (BD Bioscience, USA). Histograms were built using BD FACSDiva 8 software (BD Bioscience, USA).

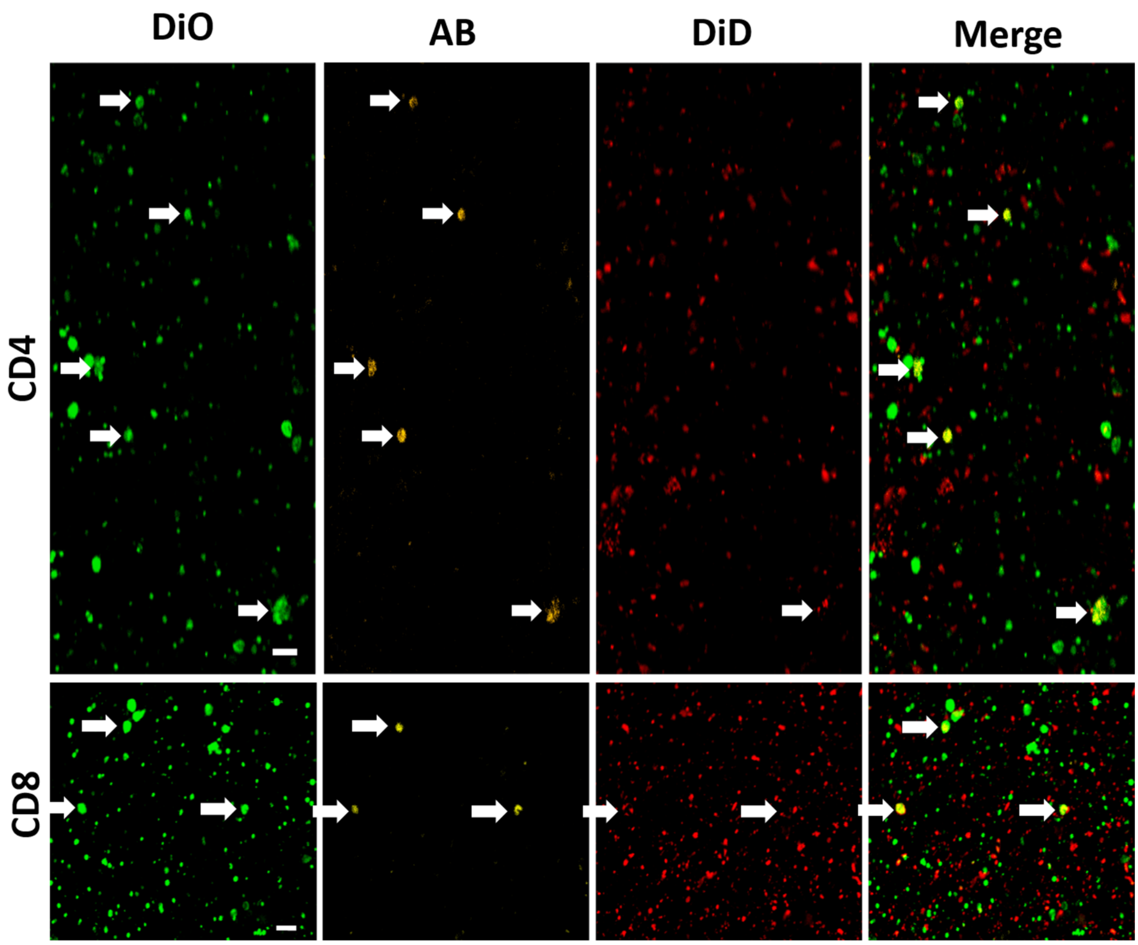

Figure 4. Analysis of co-expression of DiD-stained CIMVs with CD4-positive and CD8-positive cell populations. ⇨ co-localization of DiD and AB-staining signals. Scale bar 20 μm.

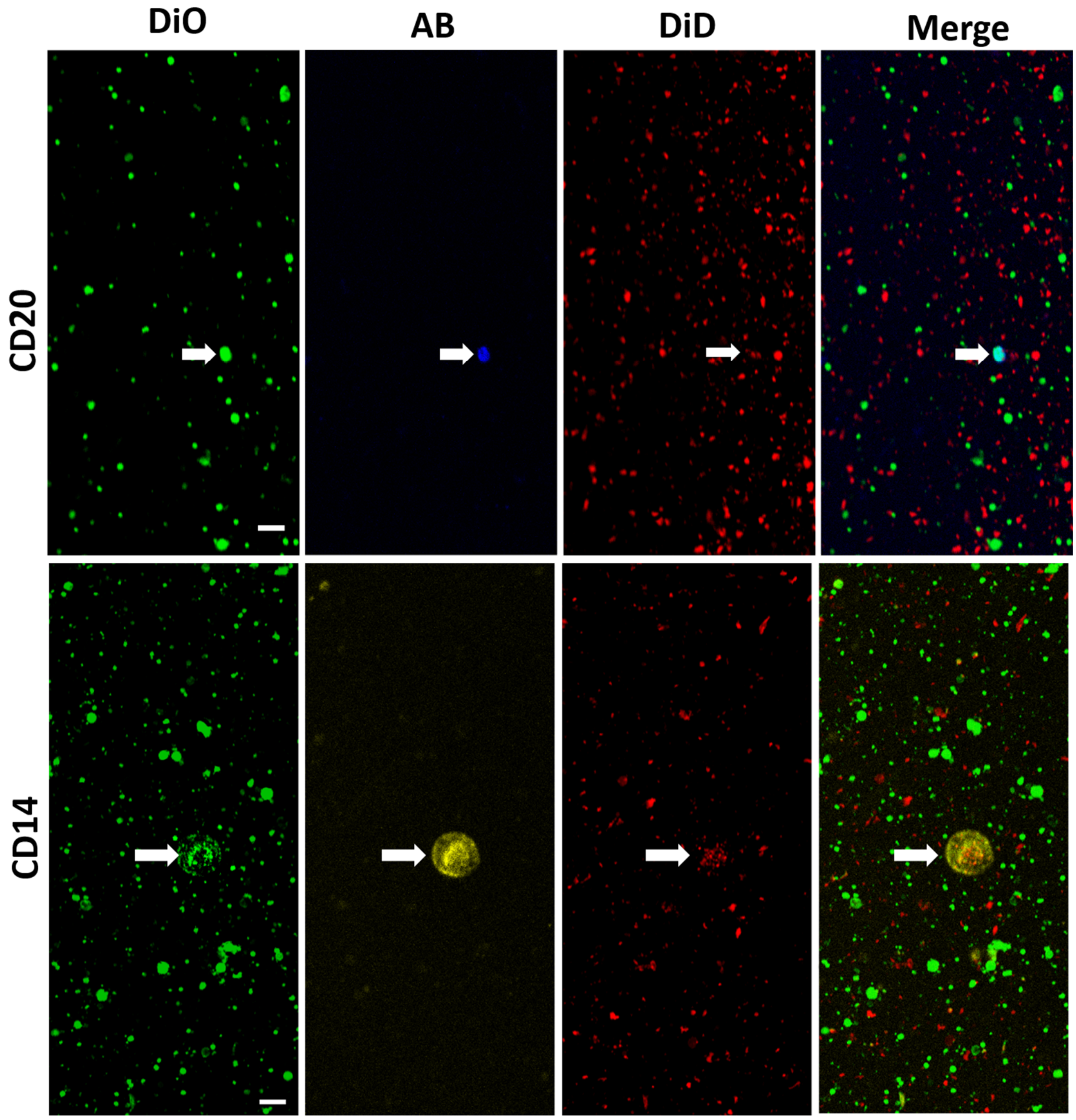

Figure S5. Analysis of co-expression of DiD-stained CIMVs with CD20-positive and CD14-positive cells populations. ⇨ co-localization of DID and AB-staining signals. Scale bar 20 μm.
